# Identification of genes related to chlamydospore formation in *Clonostachys rosea* 67‐1

**DOI:** 10.1002/mbo3.624

**Published:** 2018-04-10

**Authors:** Zhan‐Bin Sun, Jun Zhang, Man‐Hong Sun, Shi‐Dong Li

**Affiliations:** ^1^ Institute of Plant Protection Chinese Academy of Agricultural Sciences Beijing China

**Keywords:** chlamydospore, *Clonostachys rosea*, differentially expressed gene, reverse transcription quantitative PCR, transcriptome

## Abstract

Chlamydospores are specific structures that are of great significance to the commercialization of fungal biopesticides. To explore the genes associated with chlamydospore formation, a biocontrol fungus *Clonostachys rosea* 67‐1 that is capable of producing resistant spores under particular conditions was investigated by transcriptome sequencing and analysis. A total of 549,661,174 clean reads were obtained, and a series of differentially expressed genes potentially involved in fungal chlamydospore formation were identified. At 36 hr, 67 and 117 genes were up‐ and downregulated in *C. rosea* during chlamydospore production, compared with the control for conidiation, and 53 and 24 genes were up‐ and downregulated at 72 hr. GO classification suggested that the differentially expressed genes were related to cellular component, biological process, and molecular function categories. A total of 188 metabolism pathways were linked to chlamydospore production by KEGG analysis. Sixteen differentially expressed genes were verified by reverse transcription quantitative PCR, and the expression profiles were consistent with the transcriptome data. To the best of our knowledge, it is the first report on the genes associated with chlamydospore formation in *C. rosea*. The results provide insight into the molecular mechanisms underlying *C. rosea* sporulation, which will assist the development of fungal biocontrol agents.

## 1 INTRODUCTION

Filamentous fungi can produce two types of spores in their anamorphic stages; conidia and chlamydospores. Chlamydospores are specific structures with thick cell walls that are derived from the cytoplasm concentration of fungal hyphae or the conversion of conidia (Gow, 2002; Kang, Kim, Kim, Lee, & Lee, 2016; Martin, Douglas, & Konopka, 2005). Chlamydospores are usually produced in harsh environments such as low temperatures, unfavorable pH, and nutrient deficiency, since they are more resistant to adverse conditions (Eisman et al., 2006; Goh, Daida, & Vujanovic, 2009; Hughes, 1985). Various species of fungi can produce chlamydospores under particular conditions, including yeasts (*Candida albicans* and *C. dubliniensis*) (Citiulo, Moran, Coleman, & Sullivan, 2009; Staib & Morschhäuser, 2007), filamentous fungi (*Fusarium oxysporum*,* Trichoderma harzianum*,* Metarhizium anisopliae*, and *Ceratocystis platani*) (Baccelli et al., 2012; Bae & Knudsen, 2000; Iida, Kurata, Harimoto, & Tsuge, 2008; Li, Song, Li, & Chen, 2016; Ment et al., 2010), and macrofungi (*Cryptococcus laurentii* and *Coprinus cinereus*) (Kües, Walser, Klaus, & Aebi, 2002; Kurtzman, 1973).

As a specific propagule, chlamydospores make a distinct contribution to the life and survival of certain fungal species. Chlamydospores produced by the plant pathogenic fungi *F. oxysporum* and *Cylindrocarpon destructans* were found to survive for a long time under harsh environmental conditions, and to participate in the infection of plants as primary inocula (Couteaudier & Alabouvette, 1990; Kang et al., 2016). In human fungal pathogens such as in *C. neoformans*, chlamydospores generate infectious basidiospores (Lin & Heitman, 2005), and chlamydospores also act as diagnostic evidence in infection with *C. albicans* (Bottcher, Pollath, Staib, Hube, & Brunke, 2016; Cole, Seshan, Phaneuf, & Lynn, 1991). Chlamydospores can play a vital role in the activity of fungal biological control agents against pathogens. Chlamydospores of *Duddingtonia flagrans* can survive and germinate in animals, resulting in a reduction in parasitization by the larvae of nematodes and trematodes in host animals (Ojeda‐Robertos et al., 2009; Paz‐Silva et al., 2011). The resistant spores of *T. harzianum* and *Clonostachys rosea* have also been associated with the biocontrol of numerous plant fungal pathogens (Li, Qu, Tian, & Zhang, 2005). In some fungal species such as *Candida* and *Fusarium*, chlamydospores can be used as a taxonomic criterion (Li et al., 2012; Sancak, Colakoglu, Acikgoz, & Arikan, 2005).

Conditions for the induction of fungal chlamydospores, such as the temperature, lipopeptide inducers, and fermentation parameters, are receiving increasing attention in the field of biocontrol fungi research on species such as *Trichoderma* spp., *C. rosea* and *D. flagrans* (Federica, Alberto, Emilia, Carina, & Alfredo, 2013; Li et al., 2005). The genes associated with chlamydospore formation have been investigated, and genes encoding dolichol phosphate mannose synthase (Juchimiuk, Kruszewska, & Palamarczyk, 2015), hog1 mitogen‐activated protein kinase (Alonso‐Monge et al., 2003), transcription factors (Ghosh, Wangsanut, Fonzi, & Rolfes, 2015), the chromatin remodeling complex (Navarathna, Pathirana, Lionakis, Nickerson, & Roberts, 2016), and mitochondrial ATP‐dependent RNA helicase (Nobile, Bruno, Richard, Davis, & Mitchell, 2003) were linked to chlamydospore formation in *C. albicans*. Deletion of these genes inhibited or blocked the generation of chlamydospores. Furthermore, disruption of the sterile alpha motif domain‐encoding gene *FVS1* in *F. oxysporum* resulted in the defective development of conidiophores, but a dramatically increased chlamydospore yield, implying negative regulation of chlamydospore formation (Iida, Fujiwara, Yoshioka, & Tsuge, 2014). However, the knowledge of the genes involved in chlamydospore formation, especially in biocontrol fungi, remains limited, and the molecular mechanisms underlying chlamydospore formation are elusive.

*Clonostachys rosea* (syn *Gliocladium roseum*) is a mycoparasite and effective biocontrol agent against various plant fungal pathogens such as *Sclerotinia sclerotiorum*,* Rhizoctonia solani*, and *Botrytis cinerea* (Cota, Maffia, Mizubuti, Macedo, & Antunes, 2008; Ma, Wu, Yang, & Lu, 2004; Morandi, Maffia, Mizubuti, Alfenas, & Barbosa, 2003; Rodríguez, Cabrera, Gozzo, Eberlin, & Godeas, 2011). Previous studies revealed that *C. rosea* produced chlamydospores under specific conditions (Li et al., 2005; Sun, Chen, Liu, Li, & Ma, 2014). Compared with conidia, chlamydospores exhibit higher stress resistance, longer shelf‐life and similar control ability (Dong, Sun, Li, Peng, & Luo, 2014). However, the genes related to chlamydospore formation in *C. rosea* and their regulatory mechanisms are poorly understood. Herein, we investigated the differentially expressed genes (DEGs) in this biocontrol fungal species during chlamydospore formation and conidiation by transcriptome sequencing and analysis. The results provide a platform for further understanding the molecular mechanisms underlying chlamydospore formation, and may promote the development of highly efficient and stable chlamydospore‐producing biocontrol fungi.

## 2 MATERIALS AND METHODS

### 2.1 Strain

*Clonostachys rosea* 67‐1 was originally obtained from a vegetable garden in Hainan Province, China (Zhang, Gao, Ma, & Li, 2004) and preserved in the Agricultural Culture Collection of China (strain number: ACCC 39160).

### 2.2 Sample collection

Strain 67‐1 was incubated on potato dextrose agar (PDA) plate at 26°C for 10 days for sporulation. Spores were then eluted with 10 ml sterile distilled water and inoculated in PD broth with a concentration of 1 × 10^7^ spores·ml^−1^ on a rotary shaker at a speed of 180 r·min^−1^. After incubation at 27°C for 36 hr, the seed liquid was inoculated at a rate of 2% to produce chlamydospores in medium containing 2.5% glucose, 0.7% soybean cake powder, 0.035% urea, 0.1% K_2_HPO_4_·3H_2_O, 0.05% MgSO_4_, and 0.005% ZnSO_4_·7H_2_O in 1 L distilled water with the pH value of 6.5. The fungus was cultured at 27°C on a shaking table at a speed of 180 r·min^−1^, and the thallus was collected at 36 and 72 hr after inoculation by centrifuging at 8,000 *g* for 15 min. Samples following conidiation (control) prepared in the same culture condition, but in medium containing 0.9% glucose, 1.6% corn flour, 0.7% soybean cake powder, 0.035% urea, 0.1% K_2_HPO_4_·3H_2_O, 0.05% MgSO_4_, and 0.005% of ZnSO_4_·7H_2_O, which did not yield chlamydospores, were taken as controls. Three completely independent replicates were performed.

### 2.3 Construction of cDNA libraries and transcriptome sequencing

Total RNAs from each sample were extracted using Trizol reagent (Invitrogen, Carlsbad, CA, USA) according to the manufacturer's instruction, and residual genomic DNA was removed using DNase I (TaKaRa, Dalian, China). The concentration of RNA, and OD_260/280_ and OD_260/230_ values, were determined using a micro‐spectrophotometer (SimpLiNano, Cambridge, UK), and the RNA integrity number and 28S/18S value were determined using an Agilent 2100 Bioanalyzer (Agilent Technologies, Santa Clara, CA, USA).

mRNAs from fungal samples were enriched using magnetic beads harboring oligo (dT) and broken into short fragments using fragmentation buffer in a Thermomixer (Eppendorf, Hamburg, Germany). Reverse transcription was conducted and cDNA fragments were synthesized after purification, end reparation, addition of adenine to the 3’ end, and adapter connection. Suitable fragments were amplified to construct cDNA libraries. After quantity and quality monitoring, 12 cDNA libraries were sequenced using the Illumina HiSeq 4000 platform (Illumina Inc., CA, USA) at the Beijing Genome Institute (BGI).

### 2.4 Bioinformatic analysis of the 67‐1 transcriptome during sporulation

Raw transcriptome sequence data were filtered by removing disqualified reads containing adapters, unknown bases larger than 10%, and low‐quality sequences in which the number of bases with a quality value ≤10 was larger than 50% of the reads. Clean reads were submitted to the NCBI sequence read archive (SRA), and mapped onto the *C. rosea* 67‐1 genome (Sun, Sun, & Li, 2015) using Burrows‐Wheeler Aligner (BWA) software (Li & Durbin, 2009). These genes were further analyzed for changes in expression levels.

### 2.5 Analysis of DEGs

Transcript levels of 67‐1 during generation of the two types of spores were quantified using RNASeq by Expectation Maximization (RSEM) and calculated using the fragments per kb per million fragments (FPKM) method (Audic & Claverie, 1997). The NOISeq software package was used to screen DEGs, and genes for which expression varied more than twofold and the probability divergence was higher than 0.8 during the formation of chlamydospore were considered significantly differentially expressed compared with the control (Tarazona, Garcia‐Alcalde, Dopazo, Ferrer, & Conesa, 2011). The functions of identified DEGs were then investigated by Genome Ontology (GO) and Kyoto Encyclopedia of Genes and Genomes (KEGG) pathway enrichment analysis.

### 2.6 Validation of transcriptome data by reverse transcription quantitative PCR (RT‐qPCR)

A total of 16 DEGs were selected from the transcriptome of *C. rosea* 67‐1 for quantification under different cultural conditions using RT‐qPCR to confirm the reliability of the transcriptome data (Table 1). Primer pairs for each gene were designed using Primer Premier 6.0 software, and their specificity was verified using conventional PCR with the following program: 94°C for 3 min, 30 cycles of 94°C for 30 s, 55°C for 30 s, and 72°C for 20 s, and a final step at 72°C for 10 min.

**Table 1:** Primers used for RT‐qPCR in this study

Transcript ID	Predicted function	Primer (5’‐3’)	Product length (bp)
*Cch5729*	Catalase/peroxidase HPI	F: ATCTGGTCTGGAGGTTATCTGG	126
		R: TTTGCGACGAACTGTGGG	
*Cch43218*	Trypsin‐like protease	F: ACTTCTGCGGTGGTGTCTT	137
		R: AACGCTGACCTGAGTTCCT	
*Cch379*	Hypothetical protein	F: TCAGCCTTGGCGTAGAAG	129
		R: GGAACTCCCTCAGTAAATCG	
*Cch1803*	Glucose transporter	F: AGATGCTGGCTGACAACG	131
		R: GATGGGCAGAGGGAAGAT	
*Cch9554*	Hypothetical protein	F: ACTACCAAATCTGTGGCTCT	198
		R: CAGTCGTACTCTGGCTCAA	
*Cch21025*	Translation initiation factor	F: TACCTCCATCTCAGTCAGTTCA	177
		R: ACAGCAAAGGGACCAAGC	
*Cch295103*	Endochitinase‐like protein	F: ATGCGTTCCTCTATGGTTTCC	166
		R: CGAACAGATCGGAGGTTGC	
*Cch30431*	Ketosteroid isomerase	F: CCCTCTGCACCTTGTAATT	147
		R: ATCTTGCCGTTGTCGTTG	
*Cch30429*	Polyketide hydroxylase	F: AGATGCCCATAACCTTGC	120
		R: GCCTGATAAACGGTAAACTG	
*Cch56332*	Glycosyltransferase family 4 protein	F: TCTTGAGAATGCCGTTTG	188
		R: TGTAGAATCCCGTGTCCC	
*Cch45345*	Nitrosoguanidine resistance protein	F: TGGCTCTTGGGTCACATT	102
		R: ATCCGTATCCTCCGCATT	
*Cch30433*	Aldehyde dehydrogenase	F: TTGGTCCCGTCACAAACA	147
		R: AGCAGCAGCACCGAAGTA	
*Cch30437*	Glutathione S‐transferase	F: GCAGCATCAGAGTTCCGAATA	150
		R: TCAAACGCAGGGACAAGC	
*Cch47820*	Dihydrodipicolinate synthase	F: GCCCGAGCCCTTGTATGA	198
		R: AGGAGTGGTGAGCGGACAG	
*Cch30435*	Acyl esterases	F: GTCACATTGTCGCCCACT	179
		R: TGAGACTCGCTGCCATCC	
*Cch34648*	Hypothetical protein	F: AATACACGCCTCCCAATC	142
		R: GGTAATGTGCCGCTAATG	

Total RNAs extracted from thallus samples were reverse transcribed into cDNA using a cDNA FastQuant RT Kit (Tiangen, Beijing, China). The expression levels of the 16 DEGs were assayed using SYBR Premix Ex Taq II (TaKaRa, Dalian, China) with *actin* and *succinate‐semialdehyde dehydrogenase* (*SSD*) serving as internal reference genes (Zhang, Sun, Li, & Sun, 2017) on a IQ 5 multicolor real‐time PCR detection system (Bio‐Rad, CA, USA) in 25 μl reactions containing 1 μl of 10‐fold diluted cDNA, 1 μl of forward and reverse primer, 12.5 μl of SYBR Premix, and 9.5 μl of RNase‐free water. qRT‐PCR was performed in a 96‐well plate with the following program; 95°C for 30 s, followed by 40 cycles of 95°C 5 s and 55°C for 30 s. Fluorescence values were measured from 55°C to 95°C at 0.5°C intervals for 81 cycles to check for non‐specific amplification. The 2^−ΔΔCt^ method was used to calculate relative expression levels for all DEGs (Livak, & Schmittgen, 2001). Three replicates were performed for each reaction.

### 2.7 Statistical analysis

The expression levels of DEGs were analyzed using SAS 9.1.3 statistical software (SAS Institute Inc., Cary, NC, USA). *t* tests were used for comparisons of the means of each treatment and *p*‐values lower than 0.01 were regarded as significant.

## 3 RESULTS

### 3.1 Sporulation of *C. rosea* 67‐1

**Figure 1:**
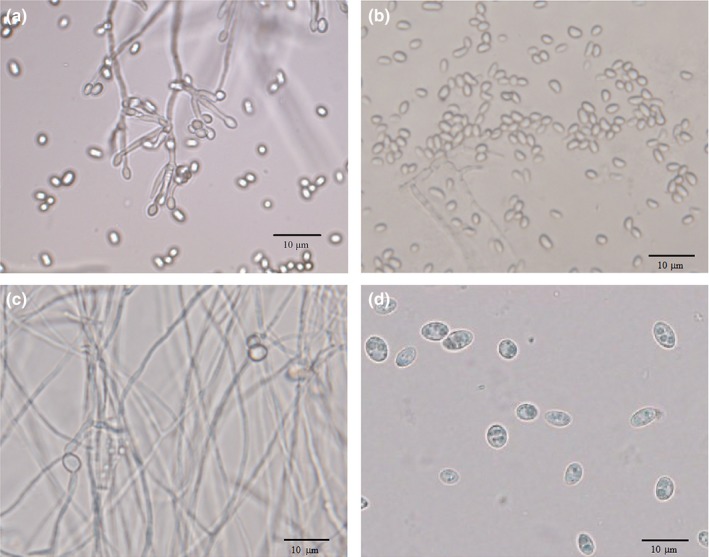
Microstructures of two types of spores produced by *C. rosea* 67‐1 growing in different media. (a) Conidia and hyphae in 36 hr. (b) Conidia in 72 hr. (c) Hyphae and newly formed chlamydospores in 36 hr. (d) Chlamydospores in 72 hr. Photos were taken at 400 × magnification. Scale bars = 10 μm

In the appropriate medium, a large amount of chlamydospores were detected after incubation for 3 days, and the generation of conidia was inhibited. Using microscopy (Olympus BX61, Tokyo, Japan), we could see that chlamydospores were derived from mycelia, either in the middle or at the tip, and most of the mycelia were transformed into chlamydospores by the end of the incubation. While in the medium for conidiation that was used as the control for transcriptome analysis of sporulation patterns in *C. rosea* 67‐1, chlamydospores were not detected (Figure 1). Compared with conidia, the resistant spores were ellipsoidal to spheroidal, 5.8 ± 0.4 × 5.0 ± 0.4 μm in size, compared to conidia (2.9 ± 0.3 × 1.6 ± 0.1 μm).

### 3.2 Bioinformatic analysis of the transcriptome

The RNA Q_20_ and Q_30_ values were above 96.1% and 90.6%, respectively, suggesting the quality and quantity of the samples met the requirements for transcriptome sequencing. All clean data were uploaded to the NCBI SRA repository with the accession numbers SRR4017358, 4017394−4017397, 4017399−4017403, 4017405, and 4017407. The average number of clean reads of the samples was 45,805,098, and each sample was mapped to the genome of *C. rosea* 67‐1 (Sun et al., 2015) with a map rate above 92.42%. The average number of expressed genes during all sampling time points was 14,755 (Table 2). Among them, unknown and metabolism‐related genes were the most highly expressed.

**Table 2:** Analysis of the transcriptome of *C. rosea* 67‐1 during sporulation

Sample^a^	Number of clean reads	Genome map rate (%)	Number of expressed genes
A36‐1	45,977,522	93.26	14,439
A36‐2	46,070,230	93.28	15,014
A36‐3	45,683,824	92.80	15,445
A72‐1	45,840,164	93.22	14,491
A72‐2	45,812,684	92.90	14,574
A72‐3	45,674,486	92.66	15,059
B36‐1	46,021,872	92.98	14,279
B36‐2	45,767,546	92.67	14,576
B36‐3	45,764,932	92.42	14,744
B72‐1	45,711,280	92.92	15,039
B72‐2	45,645,686	92.67	14,514
B72‐3	45,690,948	92.75	14,889

a Letters “A” and “B” represent the processes of conidiation (control) and chlamydospore generation in *C. rosea* 67‐1, respectively. Numbers after letters indicate the sampling time (hours), and the last numbers in sample lines refer to three completely independent replicates.

### 3.3 Analysis of DEGs

**Figure 2:**
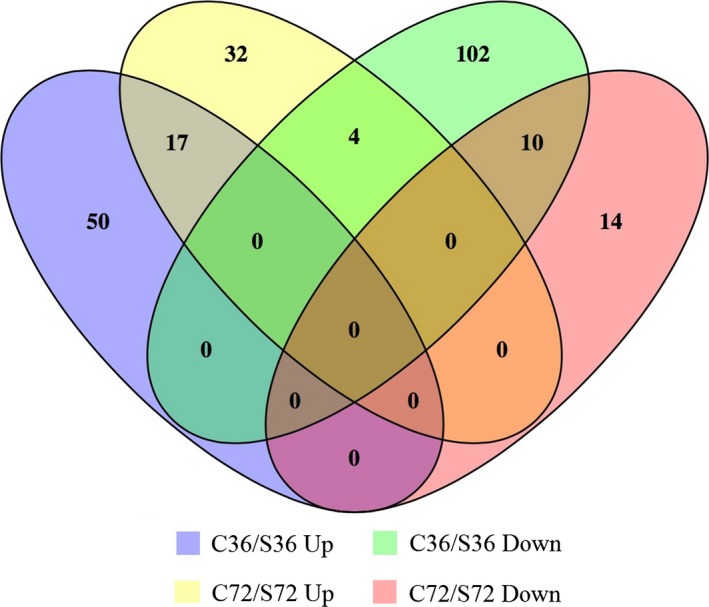
Analysis of DEGs during chlamydospore formation in *C. rosea* 67‐1. Letters “C” and “S” represent conidiation (control) and chlamydospore generation in *C. rosea* 67‐1, respectively. Numbers after letters indicate the sampling time (hrs). Up and Down refer to genes that are up‐ and downregulated during chlamydospore formation, respectively

At 36 hr, a small amount of chlamydospores were detected in the medium. Analysis of the transcriptome showed that most of genes were not differentially expressed relative to the control during chlamydospore formation, however, a total of 184 genes were differentially expressed, among which 67 were upregulated, and 117 were downregulated (Figure 2). Non‐redundant (NR) annotation indicated that 31 of the upregulated DEGs were hypothetical or predicted genes, 11 were related to metabolic processes, seven were involved in transfer functions, seven were linked to hydrolysis, and others without any annotation information were regarded as new genes. For the down‐regulated DEGs, hypothetical genes were dominant (48%), followed by new genes without known functions (24%), genes encoding metabolic process‐related enzymes (12%), genes related to transfer functions (10%), and genes linked to hydrolysis (6%). Among them, MFS transporter, glucose transporter, cytochrome P450 protein, dihydrodipicolinate synthase and salicylate hydroxylase protein had a large variation.

At 72 hr, the number of DEGs related to chlamydospore formation was lower than observed at 36 hr; 53 genes were upregulated, while only 24 were downregulated (Table S1). Among these, hypothetical genes accounted for the biggest proportion, of which 30 were upregulated and 17 were downregulated. Genes related to metabolic processes were also well represented, of which 10 were upregulated and three were downregulated. Six hydrolases encoding genes were found upregulated. At this time point, no new genes were detected.

**Figure 3:**
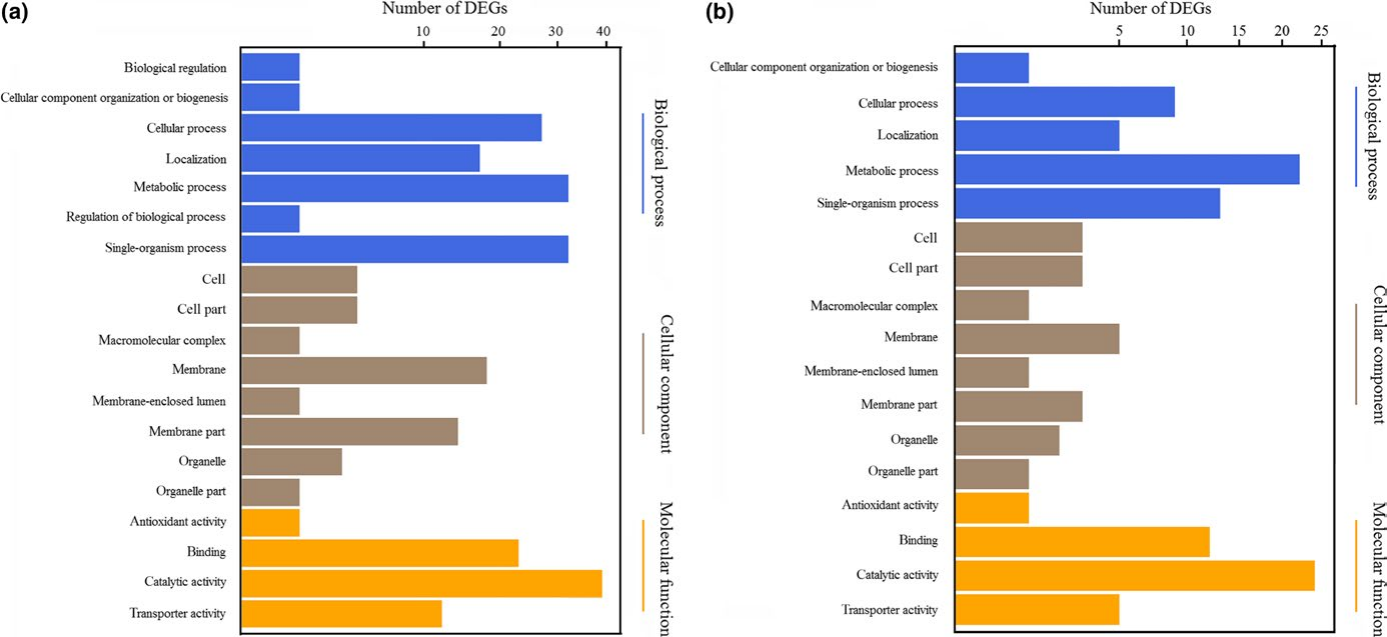
Gene ontology (GO) functional classification of differentially expressed genes during chlamydospore formation in *C. rosea* 67‐1. (a) Gene functions were divided into three categories (biological process, cellular component, and molecular function) to analyze DEGs during the early stage (36 hr) of chlamydospore generation and conidiation, during which the categories of biological regulation and regulation of biological process are specific. **(**b) Functional analysis of DEGs during the spore mass production period (72 hr)

GO functional classification of DEGs during chlamydospore formation in 67‐1 was performed, and 19 functional terms in three categories were identified at 36 hr. Metabolic process, membrane and catalytic activity were the most significant in the biological process, cellular component, and molecular function categories (Figure 3a). The dominant terms in these three categories were unaltered at 72 hr, however, biological regulation and regulation of biological process terms in the biological process category that were present during the initial stage of chlamydospore formation were not evident at this later timepoint (Figure 3b), suggesting that some signals regulating chlamydospore generation might be initiated at the early stage.

**Figure 4:**
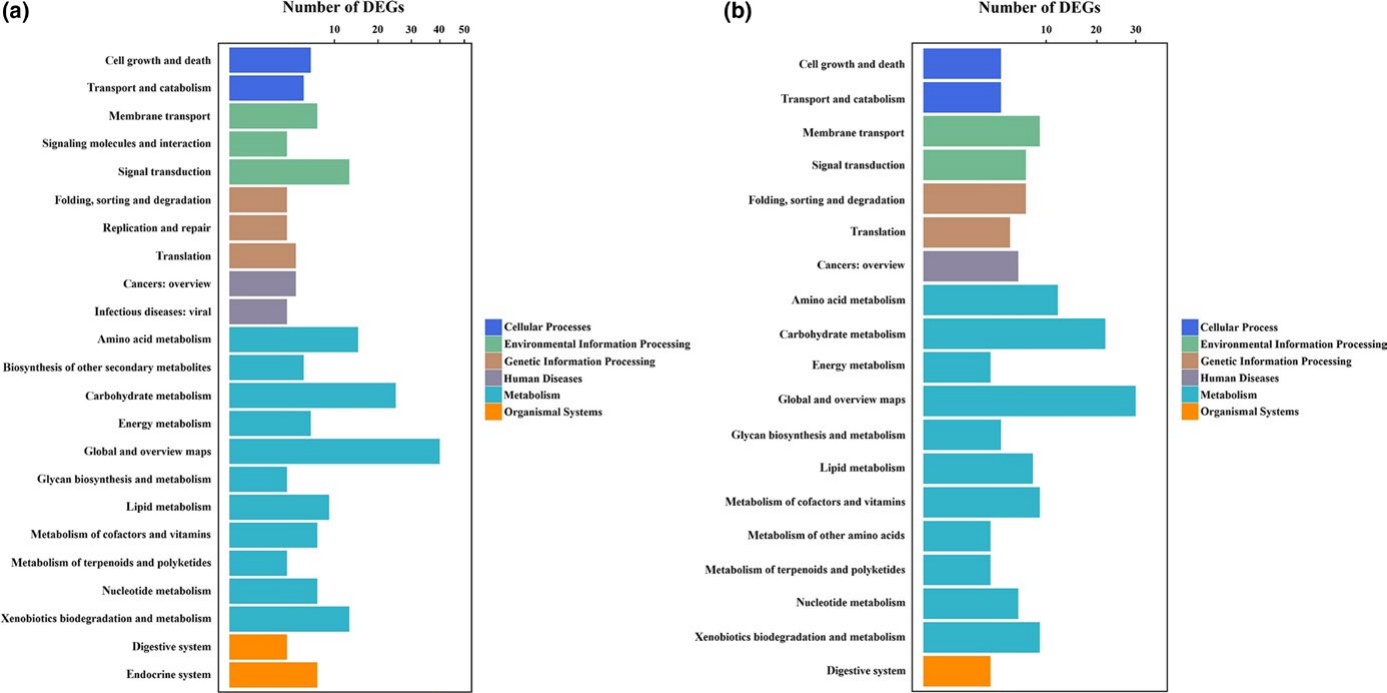
KEGG pathway analysis of DEGs during chlamydospore formation in *C. rosea* 67‐1. (a) Pathways were divided into six categories to analyze DEGs during the early stage (36 hr) of chlamydospore generation and conidiation. (b) Pathway analysis during the spore mass production period (72 hr). Different colours represent different categories of KEGG pathways; DEGs with numbers lower than 3 are not shown

A total of 188 metabolism pathways potentially involved in chlamydospore production in 67‐1 were identified using KEGG analysis. At 36 hr, 148 pathways belonging to 36 terms were found, while at 72 hr, the number of pathways was reduced to 120, covering 31 terms. Although differences existed at different timepoints, the main pathways were consistent throughout the production of chlamydospores. The term of global and overview maps in the metabolism category was dominant. Pathways involved in environmental information processing and genetic information processing were the next most abundant during the initial and stable phases of sporulation, respectively (Figure 4). The pathways associated with signaling molecules and interactions appeared at 36 hr but not at 72 hr, which also indicated that signal regulation‐related genes were essential to form chlamydospores. Most of the pathways were linked to metabolic pathways; biosynthesis of secondary metabolites and microbial metabolism in diverse environments were also detected frequently.

### 3.4 Validation of transcriptome data

**Figure 5:**
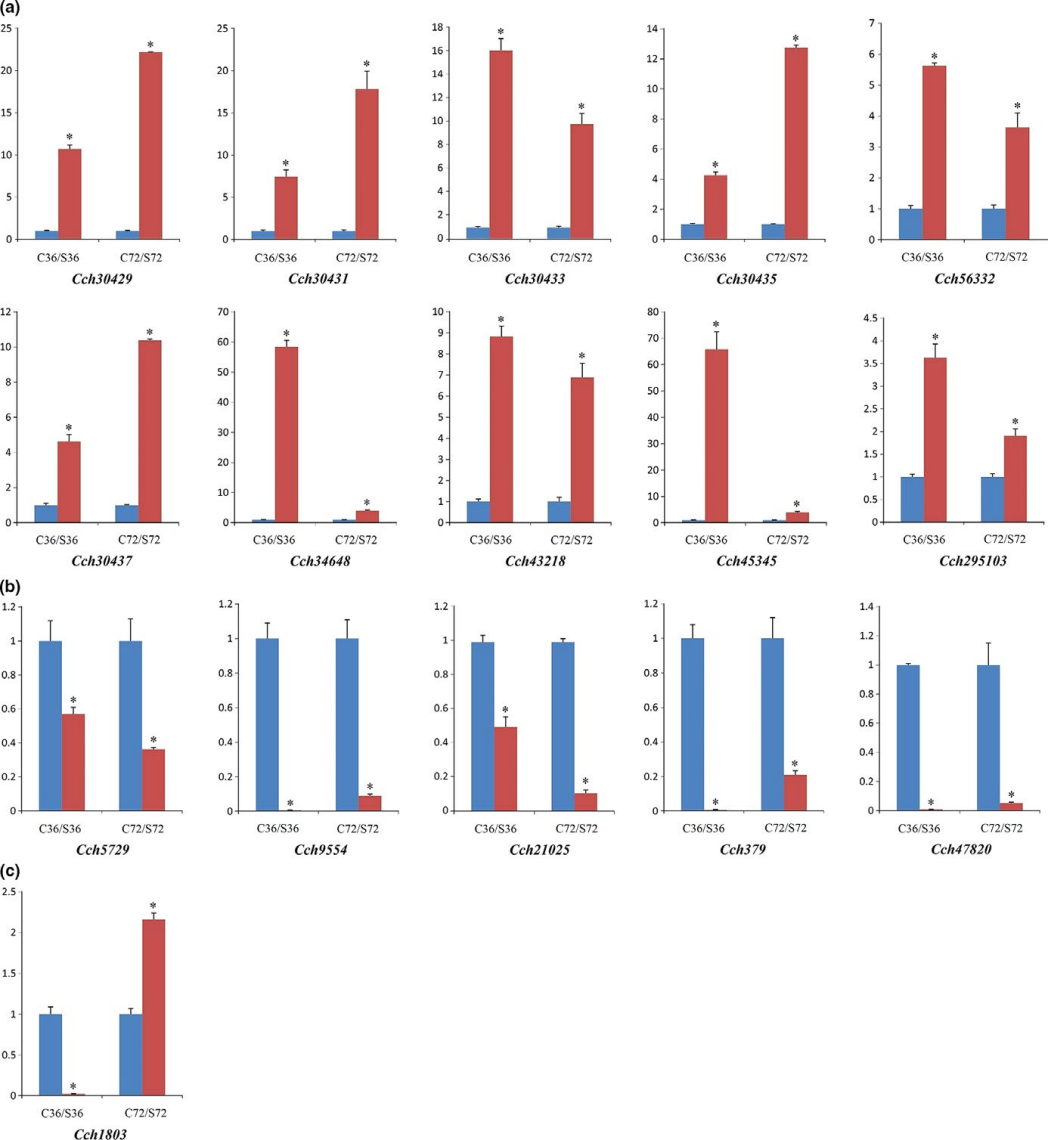
RT‐qPCR detection of 16 selected DEGs during chlamydospore formation in *C. rosea* 67‐1. (a) DEGs whose expression levels are continuously upregulated. (b) DEGs whose expression levels are continuously downregulated. (c) DEGs whose expression levels are downregulated initially, but upregulated during the later period. “C” represents controls (conidiation) and “S” represents chlamydospore generation. Numbers after letters indicate the sampling time (hours). Vertical axis represents the relative expression levels of DEGs in both sporulation processes. Error bars represent the standard deviation of three independent replicates for each tested gene, and stars on the columns represent significantly different (*p *<* *.01) according to the *t* test

Based on the results of RT‐qPCR, the expression levels of the 16 selected DEGs were divided into three groups (Figure 5). Group 1 included a large number of DEGs encoding polyketide hydroxylase, ketosteroid isomerase, glutathione S‐transferase, aldehyde dehydrogenase, nitrosoguanidine resistance protein, glycosyltransferase family 4 protein, acyl esterases, endochitinase‐like protein, trypsin‐like protease, and a hypothetical protein, the expression of which was continuously upregulated during the production of chlamydospores. In group 2, DEGs encoding a hypothetical protein, dihydrodipicolinate synthase, translation initiation factor, and catalase/peroxidase HPI were continuously downregulated. A glucose transporter‐encoding gene was placed in a third group, and was downregulated initially, but upregulated during the later stages. The expression levels of these genes were consistent with the results of transcriptome sequencing, indicating that the DEGs derived from the transcriptome during chlamydospore formation were accurate and reliable, and hence suitable for further investigation of the genes associated with chlamydospore formation in *C. rosea* 67‐1.

## 4 DISCUSSION

Chlamydospores are propagules in various fungal species that are of great significance to fungal growth and survival. Chlamydospores are highly resistant to adverse environmental conditions, and display excellent biocontrol activity for fungal biopesticides (Braga, Silva, Carvalho, Araújo, & Pinto, 2011; Maciel, Freitas, Campos, Lopes, & Araújo, 2010; Sanyal, Sarkar, Patel, Mandal, & Pal, 2008). To understand the molecular mechanisms underlying chlamydospore formation and regulation with the aim of developing biocontrol chlamydospore agents, we investigated the transcriptome of *C. rosea* 67‐1 during chlamydospore formation and compared it with the transcriptome during conidiation. Genes potentially related to the formation and development of resistant spores were subsequently identified.

In this study, chlamydospores and conidia of *C. rosea* 67‐1 were cultured in different media. In the chlamydospore‐forming medium, conidiation was greatly inhibited, while in the control medium, chlamydospores were not detected during the sampling time, and initiation of conidiation was accompanied with the development of initial hyphae during the very beginning of incubation (less than 20 hr, data not shown), which suggested that the different fungal structures could not be completely separated. Unlike in previous research on gene expression under different stress conditions and developmental stages in which conidia and mycelia were collected separately (Chen, Xie, Ye, Jensen, & Eilenberg, 2016; Llanos, François, & Parrou, 2015; Xu et al., 2015), we aimed to investigate DEGs in the biocontrol fungus *C. rosea* during chlamydospore formation by comparing with the typical developmental processes that generate conidia from mycelia. Therefore, it was not necessary to distinguish between spores and hyphae during cultivation in each condition. We harvested the thalli of 67‐1 in both media to construct *C. rosea* sporulation transcriptome therefrom.

In the transcriptome of 67‐1 during sporulation, we selected two sampling timepoints (36 hr and 72 hr). At the first sampling timepoint, a few chlamydospores were found in the medium, indicating that sporulation had switched from conidiation to chlamydospore formation. Therefore, we considered 36 hr as the initial stage of chlamydospore formation. During this stage, 67 and 117 genes were up‐ and downregulated, respectively, suggesting genes related to chlamydospore formation were activated, while those involved in conidiation were inhibited. At 72 hr, a much larger amount of chlamydospores were detected, and this timepoint was therefore considered to represent chlamydospore mass production, during which 53 genes were upregulated and 24 were downregulated. Compared to the early stage, downregulated genes were decreased more sharply. We deduced that this might be due to the marked reduction in genes expressed during conidiation (Figure 2), but this hypothesis requires further experimental verification.

Through transcriptome analysis, we identified a series of DEGs (184 at 36 hr and 77 at 72 hr) that were potentially involved in chlamydospore formation in *C. rosea*. Some of the identified DEGs encoded transporters and had a transfer function, as reported previously in *C. albicans*. Franke et al. (2006) found that deletion of the putative vesicle transport protein‐encoding gene *vac1p* affected the formation of chlamydospores. Additionally, the putative transporter gene *QDR1/CD36_29520* was confirmed to be distinctly downregulated during chlamydospore development (Palige et al., 2013). Therefore, we deduced that these genes might play roles in transporting substances needed for fungal sporulation. To date, transcription factors have attracted the most attention among the relatively few reported factors regulating chlamydospore formation. A series of genes encoding transcription factors were isolated including MAPK pathway transcription factor *cph1*, yeast‐hyphal transition transcription factor *efg1*, homeobox transcription factor *grf10* and GATA transcription factor *Gln3*, all of which were reported as key factors in chlamydospore formation and development in *C. albicans* (Bottcher et al., 2016; Ghosh et al., 2015; Maiti et al., 2015; Sonneborn, Bockmühl, & Ernst, 1999). In our present study on *C. rosea*, we also identified genes encoding transcription factors that were differentially expressed, indicating an involvement in the regulation of transcription during chlamydospore formation and development.

Among the DEGs, we also identified a number of cell wall degrading enzymes including two chitinases and a serine protease that had been previously reported in *C. rosea*. The expression of chitinase encoding gene *cr‐ech42* and *chiB2* in *C. rosea* IK726 was found to be triggered by chitin, and *cr‐ech42* was also induced by *F. culmorum* cell walls (Mamarabadi, Jensen, & Lübeck, 2008; Tzelepis, Dubey, Jensen, & Karlsson, 2015). A serine protease gene *prC* from *C. rosea* was found upregulated under some environmental stresses, for example, oxidant and heat shock, and its expression also highly increased when encountering nematode cuticle (Zou, Tao, et al., 2010; Zou, Xu, et al., 2010) . We speculate that these hydrolases might take part in the dissolve of *C. rosea* hyphae when the resistant spores release. Glycosyltransferase is an important kind of transferase and has been proved to be involved in cell wall remodeling in *Neurospora crassa* (Martínez‐Núñez & Riquelme, 2015). In our study, a glycosyltransferase encoding gene was also identified during the formation of chlamydospores. We deduced that this gene might involve in chlamydospore cell wall formation. Some genes, for example, aldehyde dehydrogenase, glucose transporter and polyketide hydroxylase that have not been reported to link with chlamydospore formation in other species were also detected, and their specific functions need further explored. A number of hypothetical genes were significantly differentially expressed as well, implying that they might be linked with interesting functions during sporulation. We determined 16 DEGs related to metabolic process, cell wall degrading enzyme and transporter using RT‐qPCR and found that their expression sharply changed in the specific condition, indicating that these genes might involve in the formation of chlamydospores in *C. rosea*. However, the functions of these DEGs in *C. rosea* during chlamydospore formation need to be further verified by gene knockout and complementation and biological assay, and the pathways involved need to be explored.

*Clonostachys rosea* is a promising biocontrol fungus active against various fungal plant pathogens, and agents from *C. rosea*, with the components of conidia and hyphae, have been registered in Europe and North America in recent decades (Gwynn, 2014). However, problems such as instability of control activity and short shelf‐life have proved a bottleneck for the industrial production and field application of *C. rosea* and other biocontrol fungi. The development of chlamydospore agents can provide effective fungal biopesticides of more consistent quality that are better suited to large‐scale applications owing to higher resistance, more stable control efficiency and longer shelf‐life. Investigation of the genes associated with chlamydospore production and determination of the molecular mechanisms underlying chlamydospore formation and regulation will greatly facilitate the development and application of *C. rosea*.

## 5 CONCLUSION

In this study, the transcriptomes of *C. rosea* during chlamydospore formation and conidiation were sequenced and analyzed, and a series of differentially expressed genes potentially involved in chlamydospore formation were identified, which were related to cellular component, biological process, and molecular function. A total of 188 metabolism pathways were suggested to be linked to chlamydospore production. To the best of our knowledge, this is the first report on genes related to chlamydospore formation in *C. rosea*. This study provides insight into the molecular mechanisms underlying *C. rosea* sporulation, and will facilitate the development of fungal biocontrol agents.

## CONFLICT OF INTEREST

None declared.
