# Supplementary material for: Identification of genes related to chlamydospore formation in Clonostachys rosea 67‐1
Source: Microbiologyopen. 2018 Apr 10;8(1):e00624. doi: 10.1002/mbo3.624 (PMC6341034; doi:10.1002/mbo3.624)
Supplement: Supplementary file 1 [file MBO3-8-e00624-s001.pdf]

Supplementary Table S1. List of DEGs in *C. rosea* 67-1 at 36 h during chlamydospore formation.

Gene ID	Up/Down regulation	Blast NR
Cch103851	up	hypothetical protein
Cch3324	up	hypothetical protein
Cch12013	up	hypothetical protein
Cch14835	up	hypothetical protein
Cch5859	up	hypothetical protein
Cch46361	up	hypothetical protein
Cch42624	up	hypothetical protein
Cch20390	up	hypothetical protein
Cch28712	up	hypothetical protein
Cch1511151	up	hypothetical protein
Cch3709	up	hypothetical protein
Cch16182	up	hypothetical protein
Cch151121	up	hypothetical protein
Cch34222	up	hypothetical protein
Cch1716	up	hypothetical protein
Cch261574	up	hypothetical protein
Cch5076	up	hypothetical protein
Cch3684	up	hypothetical protein
Cch224	up	hypothetical protein
Cch23444	up	hypothetical protein
Cch36051	up	hypothetical protein
Cch56334	up	hypothetical protein
Cch34648	up	hypothetical protein
Cch281219	up	hypothetical protein
Cch17159	up	hypothetical protein
Cch13313	up	hypothetical protein
Cch30612	up	hypothetical protein
Cch4045	up	hypothetical protein
Cch9310	up	hypothetical protein
Cch29548	up	hypothetical protein
Cch13721	up	hypothetical protein
Cch3708	up	not hit
Cch49029	up	not hit
Cch17429	up	not hit
Cch3878	up	not hit
Cch83528	up	not hit
Cch1857	up	not hit
Cch3313	up	not hit
Cch43984	up	not hit
Cch25334	up	not hit
Cch50921	up	not hit
Cch10959	up	not hit
Cch56333	up	L-lactate dehydrogenase
Cch4046	up	ACB 4-hydroxyacetophenone monooxygenase

Cch1511182	up	phosphatidylserine decarboxylase
Cch3794	up	NADH:flavin oxidoreductase
Cch10991	up	alcohol dehydrogenase 1
Cch30435	up	acyl esterases
Cch7218	up	nitroreductase family protein
Cch30433	up	aldehyde dehydrogenase
Cch45345	up	nitrosoguanidine resistance protein
Cch30429	up	polyketide hydroxylase
Cch30431	up	ketosteroid isomerase
Cch1749	up	phosphate transport protein
Cch281265	up	sugar transporter
Cch84812	up	ABC-2 type transporter
Cch56332	up	glycosyltransferase family 4 protein
Cch1304	up	H+-transporting ATPase
Cch23923	up	general substrate transporter
Cch30437	up	glutathione S-transferase
Cch67214	up	glucan endo-1,3-beta-glucosidase A1-like protein
Cch43218	up	trypsin-like protease
Cch295103	up	endochitinase-like protein
Cch230341	up	endochitinase-like protein
Cch3770	up	serine protease
Cch31832	up	serine protease
Cch16415	up	secreted serine protease protein
Cch3466	down	hypothetical protein
Cch4881	down	hypothetical protein
Cch3203	down	hypothetical protein
Cch6756	down	hypothetical protein
Cch3314	down	hypothetical protein
Cch21617	down	hypothetical protein
Cch19611	down	hypothetical protein
Cch1955144	down	hypothetical protein
Cch4399	down	hypothetical protein
Cch4522	down	hypothetical protein
Cch6751	down	hypothetical protein
Cch4392	down	hypothetical protein
Cch1575	down	hypothetical protein
Cch36012	down	hypothetical protein
Cch379	down	hypothetical protein
Cch60725	down	hypothetical protein
Cch726	down	hypothetical protein
Cch5604	down	hypothetical protein
Cch2058	down	hypothetical protein
Cch2235	down	hypothetical protein
Cch9554	down	hypothetical protein
Cch109534	down	hypothetical protein
Cch40383	down	hypothetical protein

Cch5983	down	hypothetical protein
Cch542	down	hypothetical protein
Cch29581	down	hypothetical protein
Cch5612	down	hypothetical protein
Cch169413	down	hypothetical protein
Cch32524	down	hypothetical protein
Cch723	down	hypothetical protein
Cch6597	down	hypothetical protein
Cch3485	down	hypothetical protein
Cch12641	down	hypothetical protein
Cch51041	down	hypothetical protein
Cch8691	down	hypothetical protein
Cch4065	down	hypothetical protein
Cch6933	down	hypothetical protein
Cch69027	down	hypothetical protein
Cch30823	down	hypothetical protein
Cch5511	down	hypothetical protein
Cch41720	down	hypothetical protein
Cch241141	down	hypothetical protein
Cch43951	down	hypothetical protein
Cch4461	down	hypothetical protein
Cch10826	down	hypothetical protein
Cch30917	down	hypothetical protein
Cch4509	down	hypothetical protein
Cch28154	down	hypothetical protein
Cch4389	down	hypothetical protein
Cch28015	down	hypothetical protein
Cch7624	down	hypothetical protein
Cch552	down	hypothetical protein
Cch16166	down	hypothetical protein
Cch56454	down	hypothetical protein
Cch661	down	hypothetical protein
Cch46524	down	hypothetical protein
Cch15312	down	not hit
Cch43422	down	not hit
Cch10320	down	not hit
Cch20398	down	not hit
Cch4214	down	not hit
Cch72532	down	not hit
Cch6834	down	not hit
Cch11524	down	not hit
Cch50927	down	not hit
Cch46349	down	not hit
Cch49910	down	not hit
Cch4215	down	not hit
Cch28736	down	not hit

Cch3520	down	not hit
Cch8215	down	not hit
Cch28735	down	not hit
Cch5676	down	not hit
Cch45010	down	not hit
Cch7757	down	not hit
Cch20396	down	not hit
Cch23935	down	not hit
Cch5677	down	not hit
Cch42018	down	not hit
Cch2805	down	not hit
Cch3106	down	not hit
Cch14012	down	not hit
Cch129352	down	not hit
Cch1769	down	not hit
Cch33146	down	transcriptional activator protein-like protein
Cch15311	down	zinc finger domain-containing protein
Cch553	down	timethylaniline monooxygenase
Cch2968	down	aldehyde dehydrogenase
Cch26936	down	D-2-hydroxyacid dehydrogenase-like protein
Cch26965	down	probable maltose permease
Cch8919	down	formate dehydrogenase-like protein
Cch4847	down	cytochrome p450 protein
Cch3932	down	polyphosphatidylinositol phosphatase-like protein
Cch1111	down	phosphoenolpyruvate carboxykinase
Cch17434	down	salicylate hydroxylase protein
Cch47820	down	dihydrodipicolinate synthase
Cch21025	down	translation initiation factor
Cch5729	down	catalase/peroxidase HPI
Cch18192	down	MFS transporter
Cch17433	down	MFS transporter
Cch231219	down	major facilitator superfamily transporter protein
Cch59417	down	hexose transporter-like protein
Cch59417	down	transporter-like protein
Cch11055	down	transporter-like protein
Cch3849	down	sphingoid long-chain base transporter-like protein
Cch34341	down	sphingoid long-chain base transporter-like protein
Cch2086	down	methyltransferase
Cch2081	down	glutamyl-tRNA(gln) amidotransferase subunit A
Cch1803	down	glucose transporter
Cch1231	down	glucose transporter rco-like protein
Cch396	down	chitinase
Cch129137	down	glucan 1,4-alpha-glucosidase
Cch4866	down	glycoside hydrolase family 93
Cch1725	down	N-acetyl-beta-D-glucosaminidase
Cch56481	down	beta-glucosidase A-like protein

Cch34520	down	endo-1,3(4)-beta-glucanase
Cch9226	down	endo-1,3(4)-beta-glucanase
