# Supplementary material for: Identification of genes related to chlamydospore formation in Clonostachys rosea 67‐1
Source: Microbiologyopen. 2018 Apr 10;8(1):e00624. doi: 10.1002/mbo3.624 (PMC6341034; doi:10.1002/mbo3.624)
Supplement: Supplementary file 2 [file MBO3-8-e00624-s002.pdf]

Supplementary Table S2. List of DEGs in *C. rosea* 67-1 at 72 h during chlamydospore formation.

Gene ID	Up/Down regulation	Blast NR
Cch30436	up	hypothetical protein
Cch3537	up	hypothetical protein
Cch58516	up	hypothetical protein
Cch719	up	hypothetical protein
Cch34221	up	hypothetical protein
Cch4399	up	hypothetical protein
Cch11129	up	hypothetical protein
Cch9213	up	hypothetical protein
Cch24195	up	hypothetical protein
Cch34222	up	hypothetical protein
Cch260211	up	hypothetical protein
Cch5672	up	hypothetical protein
Cch5419	up	hypothetical protein
Cch5497	up	hypothetical protein
Cch103898	up	hypothetical protein
Cch30434	up	hypothetical protein
Cch3121	up	hypothetical protein
Cch186138	up	hypothetical protein
Cch23444	up	hypothetical protein
Cch26233	up	hypothetical protein
Cch29577	up	hypothetical protein
Cch30428	up	hypothetical protein
Cch50412	up	hypothetical protein
Cch2959	up	hypothetical protein
Cch30432	up	hypothetical protein
Cch9310	up	hypothetical protein
Cch29548	up	hypothetical protein
Cch34648	up	hypothetical protein
Cch12556	up	hypothetical protein
Cch34515	up	hypothetical protein
Cch30429	up	polyketide hydroxylase
Cch30431	up	ketosteroid isomerase
Cch30433	up	aldehyde dehydrogenase
Cch30435	up	acyl esterases
Cch45345	up	nitrosoguanidine resistance protein
Cch103896	up	D-xylose 1-dehydrogenase-like protein
Cch6912	up	glucosamine-6-phosphate deaminase
Cch6913	up	hexokinase-like protein
Cch80413	up	cholesterol dehydrogenase
Cch1757	up	acid phosphatase
Cch1803	up	glucose transporter
Cch56332	up	Glycosyltransferase family 4 protein

Cch30437	up	Glutathione S-transferase
Cch3849	up	Sphingoid long-chain base transporter-like protein
Cch1749	up	phosphate transport protein
Cch13069	up	glycosyltransferase
Cch281265	up	Sugar transporter
Cch41860	up	endochitinase
Cch295103	up	endochitinase-like protein
Cch230341	up	endochitinase-like protein
Cch7299	up	chitinase
Cch1725	up	N-acetyl-beta-D-glucosaminidase
Cch43218	up	trypsin-like protease
Cch4818	down	hypothetical protein
Cch38215	down	hypothetical protein
Cch11518	down	hypothetical protein
Cch24177	down	hypothetical protein
Cch212215	down	hypothetical protein
Cch129137	down	hypothetical protein
Cch9242	down	hypothetical protein
Cch475	down	hypothetical protein
Cch69928	down	hypothetical protein
Cch9554	down	hypothetical protein
Cch19145	down	hypothetical protein
Cch43815	down	hypothetical protein
Cch68525	down	hypothetical protein
Cch3485	down	hypothetical protein
Cch74224	down	hypothetical protein
Cch41720	down	hypothetical protein
Cch379	down	hypothetical protein
Cch5729	down	catalase/peroxidase HPI
Cch21025	down	translation initiation factor
Cch47820	down	dihydrodipicolinate synthase
Cch59417	down	Hexose transporter-like protein
Cch1231	down	glucose transporter rco-like protein
Cch4464	down	glycoside hydrolase family 51
Cch72524	down	endo-beta-1,4-glucanase D-like protein
